# Adverse Events and Immunization Errors Following a Mass Immunization Campaignwith TAK‐003 in Dourados, Brazil: *A Post‐Marketing Safety Surveillance Analysis*

**DOI:** 10.1002/jmv.70674

**Published:** 2025-10-28

**Authors:** Luana Clemm Kuhnen Anschau, Amanda Maria Miguel Bortuluzi, Andrea da Silva Santos, Devanildo de Souza Santos, Sandra de Souza Rodrigues, Indianara Ramires Machado, Renato de Ávila Kfouri, Marco Aurélio Palazzi Sáfadi, Roberto Dias de Oliveira, Julio Croda

**Affiliations:** ^1^ Post Graduate Program in Health Sciences Federal University of Grande Dourados Dourados MS Brazil; ^2^ Health Sciences Faculty Federal University of Grande Dourados Dourados MS Brazil; ^3^ Municipal Health Department of Dourados Municipal Prefecture of Dourados Dourados MS Brazil; ^4^ Federal University of Mato Grosso do Sul Campo Grande MS Brazil; ^5^ Department of Immunizations Brazilian Society of Pediatrics São Paulo SP Brazil; ^6^ Santa Casa de São Paulo School of Medical Sciences São Paulo SP Brazil; ^7^ Nursing Course State University of Mato Grosso do Sul Dourados MS Brazil; ^8^ Oswaldo Cruz Foundation ‐ Mato Grosso do Sul Campo Grande MS Brazil

**Keywords:** adverse events, Dourados, immunization, Pharmacovigilance, Qdenga, TAK‐003

## Abstract

This descriptive observational study based on post‐marketing surveillance data evaluates adverse events following immunization (AEFI) associated with the TAK‐003 (Qdenga®) vaccine. Conducted in Dourados, Brazil, between January and November 2024, the study aimed to assess the frequency, nature, and severity of AEFI to inform public health strategies. Secondary data were obtained from the e‐SUS Notifica system, including demographic information, vaccination dates, and details of AEFI, which encompassed immunization errors and adverse events. These adverse events were classified by severity, type, and temporal distribution. Statistical analysis included incidence rates, temporal trends, and correlation analyses using SPSS software. Among 124,483 administered doses, 88 AEFI were reported, yielding an incidence rate of 70.69 per 100.000 doses. Most events (49.00 per 100.000 doses) were Nonserious, including headache, fever, and rash. Serious AEFI were rare (6.42 per 100.000 doses), with two cases of grade 1 anaphylaxis and two Guillain‐Barré syndrome (GBS) cases. Women (71.01%) and individuals aged 30–39 years (82.06 per 100.000 doses) were the most affected. Twenty seven Immunization errors were reported for 30.68% of AEFI, often linked to gaps in training. TAK‐003 demonstrated a favorable safety profile, consistent with clinical trial findings, characterized predominantly by mild, self‐limiting adverse events, with serious adverse events occurring infrequently. These findings underscore the importance of enhanced training protocols, robust surveillance systems, and timely reporting to optimize vaccine safety and maintain public trust.

## 1 Introduction

Dengue fever remains one of the most significant global public health challenges, with its burden disproportionately affecting tropical and subtropical regions, including Brazil [1, 2]. The disease, caused by four distinct viral serotypes (DENV‐1 to DENV‐4) and primarily transmitted by Aedes mosquitoes, is a leading cause of illness and hospitalization in endemic regions. Severe manifestations, such as dengue hemorrhagic fever or dengue shock syndrome, pose further threats to affected populations, exacerbating healthcare challenges in resource‐constrained settings [3, 4, 5].

Vaccination has emerged as an important strategy in mitigating the global impact of dengue [2]. Among the vaccines developed, TAK‐003 (Qdenga®) has shown promising results, with phase 3 clinical trials confirming its efficacy and safety across diverse populations. Its widespread use holds great potential for reducing dengue morbidity and mortality in endemic areas [6, 7, 8, 9]. However, post‐licensure surveillance is critical to assess vaccine safety in real‐world contexts, as clinical trials alone may not capture rare or region‐specific adverse events [10].

Vaccine safety is a global priority, and serious adverse events such as anaphylaxis, although rare, can impact public confidence and acceptance of immunization campaigns [11, 12]. In Brazil, the National Immunization Program (PNI) has established a national reporting system for adverse events supposedly attributable to vaccination, allowing for the monitoring of these events. However, data collected in routine health services do not have the same methodological rigor as randomized clinical trials. This difference is crucial, as post‐licensure surveillance data reflect practical experience and can identify patterns or risks not detected in controlled studies [13, 14].

In this context, this study aims to analyze the incidence of adverse events related to the TAK‐003 vaccine in Dourados, Mato Grosso do Sul, using data collected by the e‐SUS Notifica system. The analysis focuses on describing the incidence, frequency and severity of these events, contributing to the ongoing assessment of vaccine safety in real mass immunization scenarios. The city of Dourados was chosen as the first municipality in Brazil to implement a mass vaccination campaign with TAK‐003 in individuals aged 4‐60 years, as part of an ongoing study supported by Takeda and the Municipal Health Department, due to its significant epidemiological profile related to dengue. The TAK‐003 vaccination campaign in Dourados started in January 2024, marking the first citywide mass immunization with this vaccine in Brazil. Reported AEFIs are assessed by the State and Municipal Immunization Committees, which follow Ministry of Health guidelines for case investigation and classification [14]. Brazil has distinguished itself as the first country in the world to incorporate this vaccine into its National Immunization Program (PNI) for adolescents, with Dourados playing a key role in this process. Historically, Dourados has experienced frequent dengue outbreaks and high incidence rates, making it a strategically valuable site to assess vaccine effectiveness, safety, and logistical considerations in a real‐world context. The TAK‐003 vaccination campaign in Dourados started in January 2024, marking the first citywide mass immunization with this vaccine in Brazil. Reported AEFIs are assessed by the State and Municipal Immunization Committees, which follow Ministry of Health guidelines for case investigation and classification. Furthermore, its demographic characteristics and manageable population size provided an optimal setting for closely monitoring vaccine safety and effectiveness during the implementation phase [15].

## 2 Methods

### 2.1 Study Design and Data Source

This descriptive observational study utilized aggregated secondary data retrieved from the e‐SUS Notifica system, reflecting an ecological design based on post‐marketing surveillance, which systematically records adverse events following immunization (AEFIs). The data set covered reports from January 3, 2024, to November 8, 2024, focusing exclusively on AEFIs associated with the TAK‐003 vaccine administered in Dourados, Mato Grosso do Sul, Brazil which has an estimated population of 260,640 inhabitant [16].

AEFIs include all cases reported to e‐SUS and attributed to the TAK‐003 vaccination, encompassing both adverse events (AE) and immunization errors. An AE refers to any undesired effect occurring after vaccination, which may or may not be directly caused by the vaccine. Immunization errors involve mistakes in administering, storing, or handling the vaccine that may lead to negative outcomes.

Brazil follows internationally recognized standards for monitoring adverse events following immunization (AEFI). The Brazilian pharmacovigilance system classifies and monitors AEFIs similarly to global standards established by the World Health Organization (WHO) and the Council for International Organizations of Medical Sciences (CIOMS) [17].

### 2.2 Population and Inclusion Criteria

The study population included individuals who received at least one dose of the TAK‐003 vaccine during the specified period, with only residents of Dourados included to maintain consistency with the local vaccination campaign. Participants were excluded if they were non‐residents of Dourados/MS, received concomitant vaccinations during the study period, or had records with data inconsistencies, such as duplicate entries or registration errors.

### 2.3 Data Cleaning and Preparation

Before analysis, the data set underwent a thorough cleaning process to ensure accuracy and consistency. This process included removing duplicate entries, handling missing data by either imputing values or excluding incomplete records, verifying the correct alignment of dates, and identifying any inconsistencies in event reporting.

### 2.4 Analytical Methods

Descriptive statistics, including means, medians, and standard deviations, were used to summarize participant demographics and AEFI characteristics. Frequency and incidence rates per 100,000 doses were calculated for each event type. Temporal trends of AEFIs were analyzed to evaluate their distribution over time, while comparative analyses were conducted across age groups, sex, and vaccine doses to identify significant patterns or variations.

### 2.5 Database and Tools

The data set was generated in an Excel (.xls) file, and all personal identifiers were removed by the Municipal Health Department of Dourados/MS before it was provided to the researchers. Data analysis was performed using the Statistical Package for the Social Sciences (SPSS), version 25. Correlation analysis assessed potential relationships between vaccination timing and AEFI onset. Graphical representations, such as incidence trends and age‐specific distributions, were generated to enhance result interpretation.

### 2.6 Ethical Considerations

This study was approved by the Institutional Review Board of the State University of Mato Grosso do Sul (Opinion #7.089.420). All personal identifiers were anonymized before data access to ensure compliance with ethical and confidentiality guidelines.

## 3 Results

**Figure 1:**
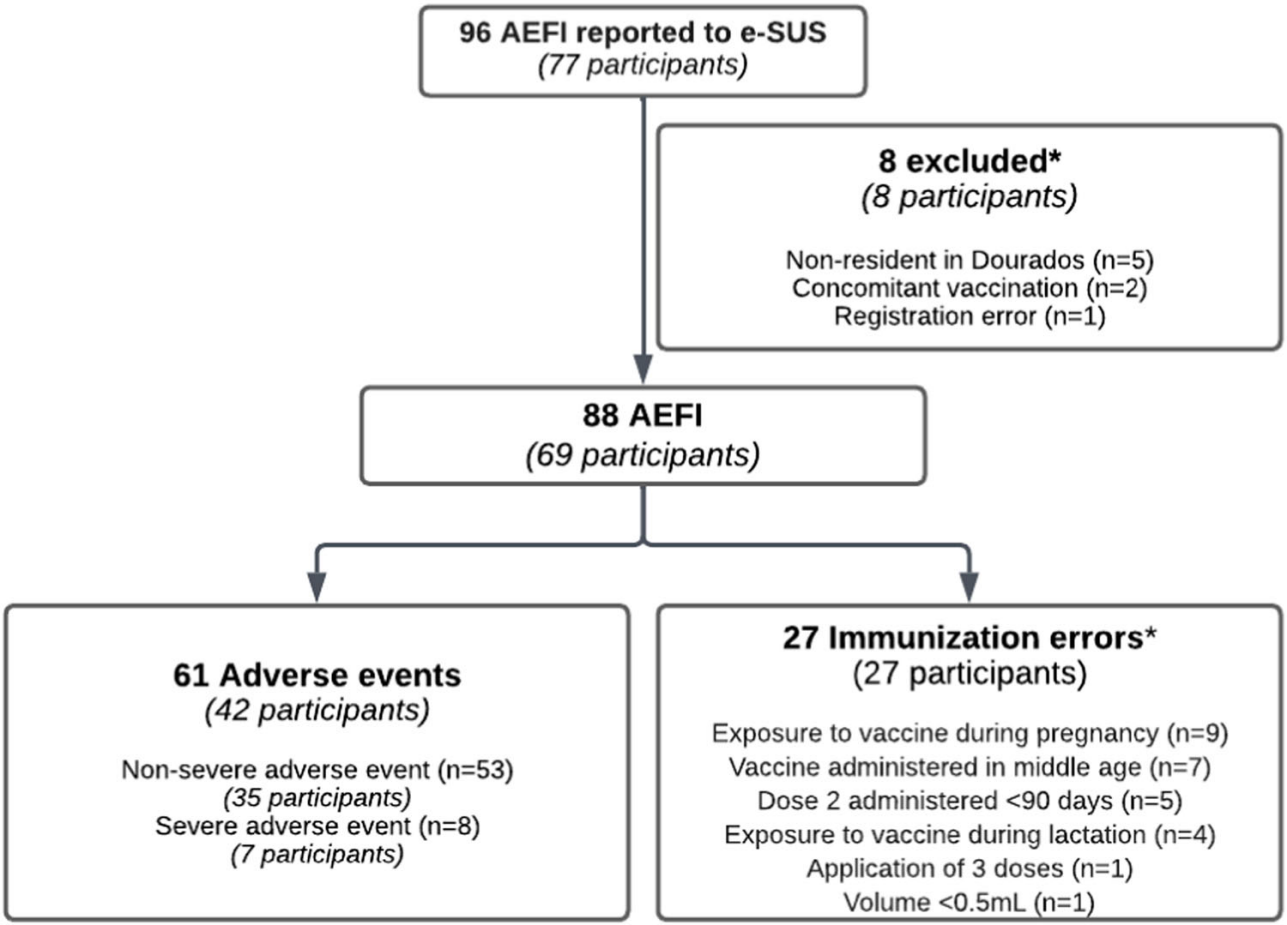
Study flowchart. *The number of AEFI is equal to the number of participants.

Seventy‐seven individuals with records of 96 AEFIs related to the TAK‐003 vaccine were identified. After processing the database, a total of 69 participants were included in the study, accounting for 88 AEFIs, with an average of 1.27 events per participant (Figure 1). Eight participants were excluded from the analysis: five were non‐residents of Dourados, two had received concomitant vaccinations, and one presented a registration error. These 88 AEFIs comprised 61 adverse events (AE) and 27 immunization errors. Overall, 124,483 vaccine doses were administered, including 89,093 first doses and 35,390 s doses. The overall incidence of AEFI was 70.69 cases per 100.000 doses, with a higher frequency observed after the first dose (84.18 per 100.000 doses, 95% CL: 67.17–105.50) and regarding the second dose (36.83 per 100,000 doses, 95% CL: 21.53–63.02, *p* < 0.001). The majority of AEFI were classified as adverse events (AE) (49.00 per 100.000 doses, 95% CL: 38.15–62.93) (Supplementary Table 1).

The average age of participants was 30.08 years (SD = 31.09; median 31; IQR 25.5), with the majority being women (71.01%, 95% CI: 59.52–80.78; *p* = 0.00048) and of white ethnicity (72.46%, 95% CI: 61.06–82.01; *p* = 0.00019) (Table 1). The age groups with the highest incidence of adverse events following immunization (AEFIs) per 100,000 doses were 30–39 years (82.06 per 100,000 doses, 95% CI: 52.55–128.10), followed by 4 to 9 years (68.42 per 100,000 doses, 95% CI: 37.18–125.90) and 50 to 59 years (62.70 per 100,000 doses, 95% CI: 35.87–109.60). Other age groups had lower incidences, such as 40–49 years (50.99 per 100,000 doses, 95% CI: 29.18–89.12) and 10 to 19 years (21.76 per 100,000 doses, 95% CI: 9.29–50.94) (Supplementary Table 2).

**Table 1:** Sociodemographic characteristics of the participants included in the study (*N* = 69).

Variable	*N*(%)
**Age,** median [IQR]	31 [25.5]
**Sex**	
Female	49 (71.01)
Male	20 (28.99)
**Race/color**	
White	50 (72.46)
Mixed	17 (24.64)
Asian	1 (1.45)
Indigenous	1 (1.45)
**Vaccine dose related to AEFI**	
First	58 (84.05)
Second	11 (15.95)
**Classification of AEFI**	
Adverse event	42 (60.87)
Immunization errors	27 (39.13)
**Time of report** *, mean (SD)	19.16 (31.09)
**Time of AEFI** **, mean (SD)	2.73 (4.71)

Abbreviations: AEFI = Adverse Event following Immunization, SD = Standard deviation.

* Days elapsed between the onset of the event and the report to the surveillance service

** *N* = 64 participants. Days elapsed between vaccination and the onset of the event.

The temporal distribution of AEFIs showed a decreasing trend over the period. The highest incidence of Nonserious events was recorded in January (125,74 cases per 100.000 doses). In contrast, serious events showed two peaks: the first in February (16,81 cases per 100.000 doses) and the second in June (10,17 cases per 100.000 doses). The distribution of immunization errors peaked in January (34,29 cases per 100.000 doses) and then stabilized, with an average incidence of 17.75 cases per 100.000 doses.

Of the participants (60.87, 95% CL: 49.02–71.83) experienced approximately 2.73 days (SD = 4.71) after receiving the vaccine. The mean time between the onset of AEs and reporting to the surveillance system was 19.16 days (SD = 31.09) (Table 1). On average, the AEFIs lasted 3.09 days (SD = 18.0), with a minimum duration of 1 day and a maximum of 17 days.

The most frequent local events were erythema (5.23 cases per 100,000 doses; 95% CL: 2.72–11.61), pruritus (4.81 cases per 100,000 doses, 95% CL: 2.20–10.52), and urticaria (2.40 cases per 100,000 doses, 95% CL: 0.81–7.08) (Supplementary Table 3).

Among the AE (63.03%, 95% CI: 51.39–74.83) were systemic, with the highest incidence per 100,000 doses observed for headache (4.81 cases per 100,000 doses, 95% CI: 2.20–10.52), fever (4.01 cases per 100,000 doses, 95% CI: 1.71–9.40), and rash (0.32 cases per 100,000 doses, 95% CI: 0.12–0.82) (Supplementary Table 3). Two cases of postvaccination dengue were confirmed, one of which was caused by a serotype not circulating in the studied region [18]. Unexpected AEs, such as Guillain‐Barré Syndrome (GBS), nausea, vomiting, diarrhea, and paresthesia, were also reported.

Most AEs were classified as Nonserious (42.57, 95% CL: 32.56–55.68) (Table 2). There were eight serious AEs (6.42, 95% CL: 3.25–12.68), including two cases of grade 1 anaphylaxis in children, according to the revised Brighton Collaboration classification [19]. The incidence of anaphylaxis was 1.60 cases per 100,000 doses administered (95% CL: 0.44–5.85).

**Table 2:** Types of Adverse Events (AEs) and Immunization Errors (IEs) (*N* = 61 AEs, *N* = 27 IEs).

Events	*N*	%	Incidence*	IC95%
**Nonserious**				
Erythema	7	11.47	5.62	2.72–11.61
Headache	6	9.83	4.81	2.20–10.52
Pruritus	6	9.83	4.81	2.20–10.52
Petechiae	4	6.55	3.21	1.25–8.26
Fever	4	6.55	3.21	1.25–8.26
Fatigue	3	4.91	2.40	0.81–7.08
Gait Disturbance	3	4.91	2.40	0.81–7.08
Urticaria	3	4.91	2.40	0.81–7.08
Abdominal Pain	2	2.89	1.60	0.44–5.85
Dengue Fever	2	2.89	1.60	0.44–5.85
Myalgia	2	2.89	1.60	0.44–5.85
Nausea and Vomiting	2	2.89	1.60	0.44–5.85
Diarrhea	1	1.44	0.80	0.14–4.55
Syncope	1	1.44	0.80	0.14–4.55
Facial Edema	1	1.63	0.80	0.14–4.55
Skin Hardening	1	1.63	0.80	0.14–4.55
Localized Edema	1	1.63	0.80	0.14–4.55
Ecchymosis	1	1.63	0.80	0.14–4.55
Warmth	1	1.63	0.80	0.14–4.55
Papules	1	1.63	0.80	0.14–4.55
Injection Site Pain	1	1.63	0.80	0.14–4.55
**Serious**				
Anaphylaxis grade 1	2	2.89	1.60	0.44–5.85
Guillain‐Barré Syndrome	2	2.89	1.60	0.44–5.85
Systemic Arterial Hypertension (SAH)	1	1.63	0.80	0.14–4.55
Facial Edema	1	1.63	0.80	0.14–4.55
Paresthesia	1	1.63	0.80	0.14–4.55
Fever	1	1.63	0.80	0.14–4.55
**Immunization errors**				
Vaccine exposure during pregnancy	9	33.33	7.22	3.80–13.74
Vaccine administered at inappropriate age	7	25.92	5.62	2.72–11.61
Second dose administered < 90 days	5	18.51	4,01	1.71–9.40
Vaccine exposure during lactation	4	14.81	3.21	1.25–8.26
Administration of 3 doses	1	3.70	0.80	0.14–4.55
Volume < 0,5 mL	1	3.70	0.80	0.14–4.55

Abbreviation: CL = confidence level.

* Incidence rate per 100.000 administered doses.

The first case of anaphylaxis involved a 5‐year‐old female child who received the vaccine on January 15, 2024. She presented anaphylaxis 20 min after the vaccine was administered, exhibiting cough, difficulty breathing, facial edema, abdominal pain, and disseminated maculopapular rash. Her condition rapidly worsened 5 min into care at the primary health care unit. She was administered 0,5 ml of adrenaline IM and 4 doses of salbutamol 100 mg, and was urgently referred to the hospital unit, where she was observed and discharged on the same day without sequelae. The second case involved a 4‐year‐old male child who received the vaccine on February 1, 2024. He presented anaphylaxis 10 min after the vaccine was administered, with coughing, sneezing, facial edema, and conjunctival hyperemia. He received immediate medical attention at the Primary Health Units and was administered 0.5 ml of adrenaline IM and 3.5 vials of hydrocortisone ‐ sodium succinate 100 mg. He was then transferred to the emergency care unit and discharged after 24 h, recovering without sequelae.

The first reported case of GBS occurred in a 16‐year‐old male adolescent who was vaccinated on March 23, 2024, and presented with fever, headache, retro‐orbital pain, nausea, dizziness, dyspnea, low back pain (L3–L5), tetraparesis, and lower limb paresthesia 24 h later. Initially hospitalized for 2 days, he recovered movement of his upper limbs and was referred to the hospital for diagnostic investigation. During his hospitalization, he was prescribed dipyrone 500 mg, ondansetron 4 mg, methylprednisolone 500 mg, and omeprazole 20 mg. He was discharged on April 2, 2024, and instructed to undergo outpatient follow‐up, which he did with good recovery. The second case of GBS involved a 40‐year‐old man who received the second dose of the vaccine on June 5, 2024 (with the first dose administered on March 2, 2024). Nineteen days after vaccination the second dose, he presented with flaccid tetraparesis, worsening finger strength, greater loss of strength in the left hand, impaired foot flexion, and previous bradycardia. He was admitted to the critical care unit on June 27, 2024, where he remained for 5 days for treatment with immunoglobulin and monitoring of adverse reactions. He was discharged after treatment and recovered without sequelae.

Immunization errors accounted for 30.68% of the AEFI (21.68, 95% CL: 14.91–31.56). The most common errors were the vaccination of pregnant women (7.22, 95% CL: 3.80–13.74), administration of the vaccine outside the recommended age range (5.62, 95% CL: 2.72–11.61), and application of the second dose at an interval of less than 90 days (4.01, 95% CL: 1.71–9.40) (Table 2).

## 4 Discussion

The study confirms that the Qdenga® vaccine (TAK‐003) has a favorable safety profile, with most AEs being mild and transient in the context of mass vaccination campaigns. Adverse events peaked at the beginning of the vaccination campaign decreased over time. Most AEFI reported were nonserious, including headache, fever, and rash. Serious AEs were rare but included 2 grade 1 anaphylaxis in children and 2 GBS. This study highlights the critical role of robust post‐approval pharmacovigilance systems in vaccine safety, emphasizing their necessity for identifying rare adverse events.

The incidence of AEFI was 70.69cases per 100.000 doses, with a higher incidence after the first dose, (84.18, 95% CL: 67.17–105.50). Among participants (60.87%, 95% CL: 49.02–71.83) experienced AEs approximately 2.73 days after vaccination. This reactogenicity may be linked to an intense initial immune response and immune system activation [7, 20, 21].

The temporal distribution of AEFI showed a decreasing trend, peaking in January (154.32 cases per 100.000 doses), likely due to the start of the vaccination campaign [22]. In January, 24 adverse events and 6 immunization errors were recorded. Robust surveillance systems are crucial for early identification and management of adverse events and immunizations errors [8, 23]. The average duration of nonserious AEs was brief, self‐limiting, and resolved without intervention, consistent with findings from other vaccine studies that also reported predominantly mild and transient events [14, 24, 25]. The rapid resolution reinforces the favorable safety profile of the TAK‐003 vaccine, as most AEs are temporary and short‐lived [7, 14, 26, 27, 28].

Serious adverse events (SAE) were uncommon, with an overall incidence of 6.42 per 100,000 (95% CI: 3.25–12.68). Among these, two cases of grade 1 anaphylaxis occurred in children aged 4 and 5 years, corresponding to an incidence of 1.6 cases per 100,000 doses. This observed rate is lower than the 5.8 cases per 100,000 doses reported by the Brazilian Ministry of Health following TAK‐003 administration [24, 29, 30]. The variation in incidence rates between our findings and the national data likely reflects differences in study populations, surveillance methodologies, and reporting sensitivity. Notably, our study population spanned a wider age range (4–59 years), while the Brazilian national vaccination program prioritized children and adolescents, in some municipalities, at the initial phase of vaccine deployment. This AE should be disclosed in the vaccine package insert but does not contraindicate use in high‐burden areas [19, 31]. The Brazilian Ministry of Health recommends that TAK‐003 vaccination be carried out exclusively in Basic Health Units [32]. Ensuring that vaccination is carried out in environments where healthcare professionals are trained to recognize and manage anaphylaxis can effectively mitigate the risks associated with these rare but serious events. The risk of anaphylaxis, while a serious consideration, does not outweigh the substantial public health benefits of TAK‐003 vaccination in dengue‐endemic areas. Dengue remains a major public health concern in Brazil and other tropical regions, contributing substantially to morbidity and mortality. The implementation of an effective vaccination strategy has the potential to markedly reduce the overall disease burden, decrease hospitalization rates, and mitigate the strain on healthcare systems.

The occurrence of unexpected SAE like GBS, nausea, vomiting, diarrhea, and paresthesia highlights the importance of comprehensive monitoring, even with the high incidence of nonserious AEs. Cases of GBS have been reported after COVID‐19 vaccinations, especially with Oxford/AstraZeneca, Janssen, and CoronaVac vaccines [33], as well as meningococcal ACWY‐D conjugate, influenza and HPV vaccines, despite most people fully recovering from GBS. Studies show that the risk of developing GBS postvaccination is extremely low compared to the benefits of immunization and typically lower than the risk of GBS from the infections the vaccines could prevent [34]. Across COVID‐19 and influenza immunization programs, real‐world evidence consistently shows GBS to be an exceedingly rare postvaccination event. Incidence rates are on the order of 1–2 per million for most vaccines, occasionally rising to ~5–15 per million with certain vaccine platforms or during specific campaigns [35]. Our findings in a dengue vaccine rollout documented two GBS cases (incidence rate of 1.60/100.000 administered doses). Intriguingly, one of the GBS cases in our study developed symptoms just 1 day following vaccination, which, although an unusually short interval, has been reported in the literature as a possible early onset within the recognized risk window for postvaccination GBS [36].

The relationship between GBS and vaccination is complex and multifactorial: individual immune responses, genetic, and environmental factors can influence its occurrence [37, 38, 39, 40]. Vaccination during seasonal periods complicates distinguishing between GBS cases caused by infection or the vaccine, creating limitations in surveillance and data analysis, which confounds the assessment of underlying causes [41]. This perspective affirms the importance of vaccination programs, alongside vigilant safety monitoring, to ensure that rare adverse events like GBS are continually assessed against their proven public health benefits.

Among the reported AEs, 63.03% (95% CL 51.39–74.83) were systemic. Beyond the expected events described in clinical trials, there were two cases of postvaccination dengue fever, with the serotype found in the patient identified as type 4, which is not circulating in the studied region [18]. This underscores the importance of monitoring dengue occurrence in vaccinated individuals, particularly in areas with different circulating dengue serotypes [42].

About one‐third of reported AEFI (30.68%; 95% CL: 21.73–40.89) were classified as immunization errors (21.68, 95% CL: 14.91–31.56), often due to lack of training, supervision, and pressures of mass vaccination campaigns. Notable errors included vaccinating pregnant women, administering vaccines outside the recommended age range and giving the second dose too early. Errors increase during large‐scale campaigns and are mainly related to incorrect vaccine administration [43, 44]. These errors can compromise immunization effectiveness and increase AE risks. Decision support technologies like alert systems and reminders in electronic medical records, along with advanced data analysis techniques such as (natural language processing) NLP, can help minimize errors and improve adherence to vaccination guidelines [43, 45, 46].

Despite the relevance of the findings, the study has limitations, including data quality concerns, and reporting bias. Secondary data may vary in accuracy and completeness, leading to underreported or misclassified adverse events (passive surveillance). Retrospective data collection is prone to errors and inconsistencies, and unmeasured confounding variables can distort the relationship between Qdenga vaccination and adverse events. Additionally, these studies cannot establish causality as robustly as prospective ones and are limited to identifying associations. The e‐SUS notifica database may lack detailed information, affecting control of confounding factors, such as in GBS cases where both vaccination and dengue infection may be associated. Reporting bias is another issue, as mild AE may be underreported.

## 5 Conclusions

This study confirms the acceptable safety profile of the TAK‐003 vaccine, with mild adverse events being the most frequently reported and serious adverse events remaining rare. Underreporting of adverse events, especially mild or nonserious ones, remains a challenge, emphasizing the need for more robust reporting systems and continuous training of health professionals to ensure effective postvaccination monitoring. Moreover, administration errors highlight the need for better training of vaccinators and improved immunization protocols.

## Author Contributions

The conception and design of the study, or acquisition of data, or analysis and interpretation of data: Luana Clemm Kuhnen Anschau, Roberto Dias de Oliveira, Andrea da Silva Santos, Devanildo de Souza Santos, Sandra de Souza Rodrigues, Indianara Ramires Machado, and Julio Croda; Drafting the article or revising it critically for important intellectual content: Luana Clemm Kuhnen Anschau, Roberto Dias de Oliveira, Julio Croda, and Andrea da Silva Santos; Final approval of the version to be submitted: Luana Clemm Kuhnen Anschau, Amanda Maria Miguel Bortuluzi, Andrea da Silva Santos, Devanildo de Souza Santos, Sandra de Souza Rodrigues, Indianara Ramires Machado, Renato de Ávila Kfouri, Marco Aurélio Palazzi Sáfadi, Roberto Dias de Oliveira, and Julio Croda.

## Conflicts of Interest

J.C. reports a relationship with Takeda Pharmaceutical Company Limited that includes: consulting, advisory and research grants. M.A.P.S. reports research grants and personal fees for advisory boards from Takeda and Sanofi. R.A.K. reports personal fees for advisory boards from Takeda and Sanofi. The other authors declare that they have no known competing financial interests or personal relationships that could have appeared to influence the work reported in this paper.

## Supporting information


**Supplementary Table 1:** Incidence rate of AEFI after dose 1 (D1), dose 2 (D2) and both (D1 and D2). **Supplementary Table 2:** AEFI's* incidence according to age group (N = 61). **Supplementary Table 3:** Adverse event classification, proportion, and incidence rate (N = 61).

## Data Availability

Data sharing not applicable to this article as no datasets were generated or analysed during the current study.
