# Supplementary material for: Adverse Events and Immunization Errors Following a Mass Immunization Campaignwith TAK‐003 in Dourados, Brazil: A Post‐Marketing Safety Surveillance Analysis
Source: J Med Virol. 2025 Oct 28;97(11):e70674. doi: 10.1002/jmv.70674 (PMC12560204; doi:10.1002/jmv.70674)
Supplement: Supplementary file 1 — Supplementary Table 1: Incidence rate of AEFI after dose 1 (D1), dose 2 (D2) and both (D1 and D2). Supplementary Table 2: AEFI's* incidence according to age group (N = 61). Supplementary Table 3: Adverse event classification, proportion, and incidence rate (N = 61). [file JMV-97-e70674-s001.docx]

**Supplementary Table**

**Supplementary Table 1** - Incidence rate of AEFI after dose 1 (D1), dose 2 (D2) and both (D1 and D2)

**Supplementary Table 2** - AEFI’s* incidence according to age group (N = 61)

**Supplementary Table 3** - Adverse event classification, proportion, and incidence rate (N = 61).

**Supplementary Table 1**

| **Occurrence** | **N** | **Incidence*** | **CL95%** |
| --- | --- | --- | --- |
| **AEFI D1** | 75 | 84.18 | 671.70–1055.00 |
| Adverse event D1 | 55 | 61.73 | 474.40–803.40 |
| Immunization errors D1 | 20 | 22.44 | 145.30–346.70 |
| **AEFI D2** | 13 | 36.73 | 214.70–628.40 |
| Adverse event D2 | 6 | 169.53 | 77.71–369.9 |
| Immunization errors D2 | 7 | 197.79 | 95.82–408.20 |
| **AEFI D1 and D2** | 88 | 706.92 | 573.90–870.80 |
| Adverse event D1 and D2 | 61 | 490.02 | 381.50–629.30 |
| Immunization errors D1 and D2 | 27 | 216.89 | 149.10–315.60 |

*Incidence rate per one million administered doses.

**Abbreviations:** AEFI = adverse event following immunization, CL = confidence level, D1 = first dose, D2 - second dose.

**Supplementary Table 2**

| **Age group** | **N** | **%** | **CL95%** | **Incidence**** | **CL95%** |
| --- | --- | --- | --- | --- | --- |
| 4–9 | 10 | 16.39 | 9.15–27.61 | 68.42 | 37.18–125.90 |
| 10–19 | 5 | 8.20 | 3.55–17.79 | 21.76 | 9.29–50.94 |
| 20–29 | 3 | 4.92 | 1.68–13.49 | 13.67 | 4.65–40.19 |
| 30–39 | 19 | 31.15 | 20.94–43.59 | 82.06 | 52.55–128.10 |
| 40–49 | 12 | 19.67 | 11.63–31.31 | 50.99 | 29.18–89.12 |
| 50–59 | 12 | 19.67 | 11.63–31.31 | 62.70 | 35.87–109.60 |

*Only AEFIs classified as adverse events, regardless of severity, were included.

**Incidence rate per 100,000 administered doses.

**Abbreviations:** Adverse Event following Immunization, CL = confidence level.

**Supplementary Table 3**

| **Events** | **N** | **%** | **CL95%** | **Incidence** | **CL95%** |
| --- | --- | --- | --- | --- | --- |
| **Systemic** | | | | | |
| Headache | 6 | 9.83 | 4.58–19.84 | 48.19 | 22.09–105.20 |
| Fever | 5 | 8.20 | 3.55–17.79 | 40.16 | 17.16–94.03 |
| Rash | 4 | 6.55 | 2.58–15.68 | 32.13 | 12.50–82.62 |
| Fatigue | 3 | 4.92 | 1.68–13.49 | 24.09 | 8.19–70.86 |
| Gait Disturbance | 3 | 4.92 | 1.68–13.49 | 24.09 | 8.19–70.86 |
| Anaphylaxis | 2 | 3.27 | 0.90–11.19 | 16.06 | 4.40–58.58 |
| Abdominal Pain | 2 | 3.27 | 0.90–11.19 | 16.06 | 4.40–58.58 |
| Dengue Fever | 2 | 3.27 | 0.90–11.19 | 16.06 | 4.40–58.58 |
| Facial Edema | 2 | 3.27 | 0.90–11.19 | 16.06 | 4.40–58.58 |
| Guillain-Barré Syndrome | 2 | 3.27 | 0.90–11.19 | 16.06 | 4.40–58.58 |
| Myalgia | 2 | 3.27 | 0.90–11.19 | 16.06 | 4.40–58.58 |
| Nausea and Vomiting | 2 | 3.27 | 0.90–11.19 | 16.06 | 4.40–58.58 |
| Diarrhea | 1 | 1.63 | 0.29–8.71 | 8,03 | 1.41–45.50 |
| Paresthesia | 1 | 1.63 | 0.29–8.71 | 8,03 | 1.41–45.50 |
| Syncope | 1 | 1.63 | 0.29–8.71 | 8,03 | 1.41–45.50 |
| Systemic Arterial Hypertension (SAH) | 1 | 1.63 | 0.29 - 8.71 | 8.03 | 1.41 - 45.50 |
| **Local** | | | | | |
| Erythema | 7 | 11.47 | 5.67–21.84 | 56.23 | 27.24–116.10 |
| Pruritus | 6 | 9.83 | 4.58–19.84 | 48.19 | 22.09–105.20 |
| Urticaria | 3 | 4.92 | 1.68–13.49 | 24.09 | 8.19–70.86 |
| Skin Hardening | 1 | 1.63 | 0.29–8.71 | 8.03 | 1.41–45.50 |
| Localized Edema | 1 | 1.63 | 0.29–8.71 | 8.03 | 1.41–45.50 |
| Ecchymosis | 1 | 1.63 | 0.29–8.71 | 8.03 | 1.41–45.50 |
| Warmth | 1 | 1.63 | 0.29–8.71 | 8.03 | 1.41–45.50 |
| Papules | 1 | 1.63 | 0.29–8.71 | 8.03 | 1.41–45.50 |
| Injection Site Pain | 1 | 1.63 | 0.29–8.71 | 8.03 | 1.41–45.50 |

*Incidence rate per one million administered doses.

**Abbreviations:** CL = confidence level
